# Biomarkers are used to predict quantitative metabolite concentration profiles in human red blood cells

**DOI:** 10.1371/journal.pcbi.1005424

**Published:** 2017-03-06

**Authors:** James T. Yurkovich, Laurence Yang, Bernhard O. Palsson

**Affiliations:** 1 Department of Bioengineering, University of California, San Diego, La Jolla, California, United States of America; 2 Bioinformatics and Systems Biology Program, University of California, San Diego, La Jolla, California, United States of America; 3 Department of Pediatrics, University of California, San Diego, La Jolla, California, United States of America; Chalmers University of Technology, SWEDEN

## Abstract

Deep-coverage metabolomic profiling has revealed a well-defined development of metabolic decay in human red blood cells (RBCs) under cold storage conditions. A set of extracellular biomarkers has been recently identified that reliably defines the qualitative state of the metabolic network throughout this metabolic decay process. Here, we extend the utility of these biomarkers by using them to quantitatively predict the concentrations of other metabolites in the red blood cell. We are able to accurately predict the concentration profile of 84 of the 91 (92%) measured metabolites (*p* < 0.05) in RBC metabolism using only measurements of these five biomarkers. The median of prediction errors (symmetric mean absolute percent error) across all metabolites was 13%. The ability to predict numerous metabolite concentrations from a simple set of biomarkers offers the potential for the development of a powerful workflow that could be used to evaluate the metabolic state of a biological system using a minimal set of measurements.

## Author summary

While deep-coverage omics data sets are allowing for more complete characterization of biological systems, there has been a concerted effort to identify a subset of measurements that are representative of qualitative network-level behavior. For some systems—like the human red blood cell (RBC)—such biomarkers have already been identified. Using the concentration profiles of these biomarkers as input to a statistical model, we predict quantitative concentration profiles of other metabolites in the RBC network. These results demonstrate that if good biomarkers are available for a biological system, it is possible to use these measurements to gain insight into the quantitative state of the rest of the network.

## Introduction

The data generated from deep coverage omics tools are becoming broadly available and thus their use is becoming more common [1, 2]. With this data, researchers have begun to identify metabolomics biomarkers that can be used to describe systemic behavior with only a few inexpensive and reliable measurements [3–7]. In transfusion medicine, deep coverage metabolomics data sets for human red blood cells (RBCs) in cold storage are rapidly accumulating [8] and have been used to characterize the state of the RBC metabolic network during storage [9–13].

Big data analysis of RBC metabolomics data has yielded a well-defined three-phase pattern of metabolic storage lesion that has fundamental consequences for blood storage [10, 13]. Recently, eight extracellular metabolic biomarkers have been identified that reliably define this three-phase decay process observed in RBCs [6]. These biomarkers (adenine, glucose, hypoxanthine, lactate, malate, nicotinamide, 5-oxoproline, and xanthine) recapitulate the qualitative trend of the entire metabolome. However, it has yet to be determined whether these biomarkers can be used to predict quantitative network behavior.

In this study, we determine that five of the eight biomarkers (glucose, hypoxanthine, lactate, malate, and xanthine) are not only excellent qualitative predictors, but also accurate quantitative predictors of metabolic concentrations in the rest of the metabolic network. Using a simple computational formulation [14] prevalent in a variety of fields [15–18], we extend the utility of these biomarkers by using them to quantitatively predict the concentration profiles of 91 other metabolites in the network. This added use of validated biomarkers offers the potential for a powerful workflow that utilizes five biomarkers to evaluate the state of RBC metabolism.

## Results

For this study, we used the metabolomics data set from Bordbar et al. [10] that measured 96 intracellular and extracellular metabolites in human red blood cells under storage conditions. The data set measured 14 time points over a 45 day time period for 20 biological replicates. For the purposes of modeling, we randomly divided these 20 replicates into equal sized training and testing sets of 10 samples. We observed a high amount of variability in the extracellular glucose measurement at Day 31 (S1 Fig), a behavior which was not observed in the intracellular glucose measurement (S2A Fig) but was seen in other extracellular measurements at Day 31 (S2B Fig). In order to avoid bias arising from the inclusion of potentially erroneous data, we excluded the measurements from Day 31, resulting in 13 total time points spanning 45 days of storage.

**Fig 1:**
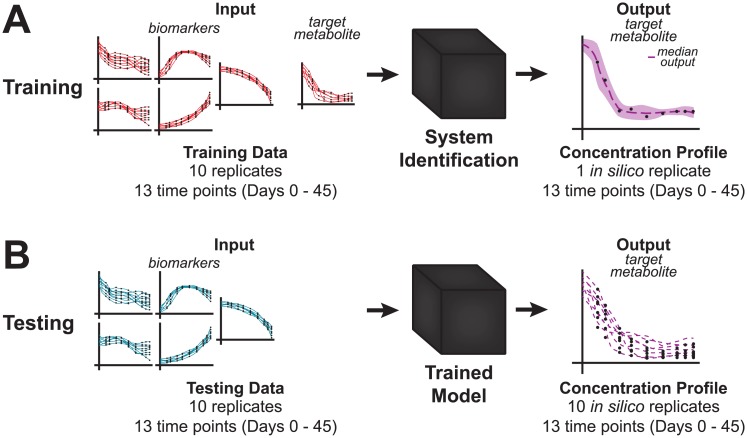
Prediction workflow. A: The model is trained on the measured concentration profiles of the five biomarkers (glucose, hypoxanthine, lactate, malate, and xanthine) and the target metabolite. B: The resulting ensemble of models (one for each replicate) can then be used to predict the concentration profile of the target metabolite given only the measured concentration profiles of the five biomarkers.

We trained multiple polynomial models of varying complexity on the concentration profiles of the biomarkers and the concentration profile of the target metabolite (Fig 1). The best performing model was a simple, linear Output-Error model [14]. Variation between blood bags is a known challenge, as both donor and technical factors contribute to sample heterogeneity [1]. Due to this variation, we noted that simply because these eight biomarkers are good qualitative predictors of systemic behavior does not imply that they are also good quantitative predictors. We therefore performed a feature selection and cross validation within the eight biomarkers, determining that adenine, nicotinamide, and 5-oxoproline were not able to quantitatively predict systemic behavior as well as the other five biomarkers (see Methods). Thus, glucose, hypoxanthine, lactate, malate, and xanthine were used for the remaining analysis.

In order to generate a prediction for each metabolite, we trained the model using the five biomarkers and a measured profile for the target metabolite as input (Fig 1). We used an ensemble modeling approach [19] to reduce bias arising from using either individual replicates or averaging replicates to train a single model. With 10 training replicates, this approach allowed us to generate an ensemble of trained models that inherently includes the biological variation of the training data (S3 Fig). We then used this trained ensemble computational model to predict a consensus concentration profile of a target metabolite, this time only using the biomarkers as input (Fig 1).

**Fig 2:**
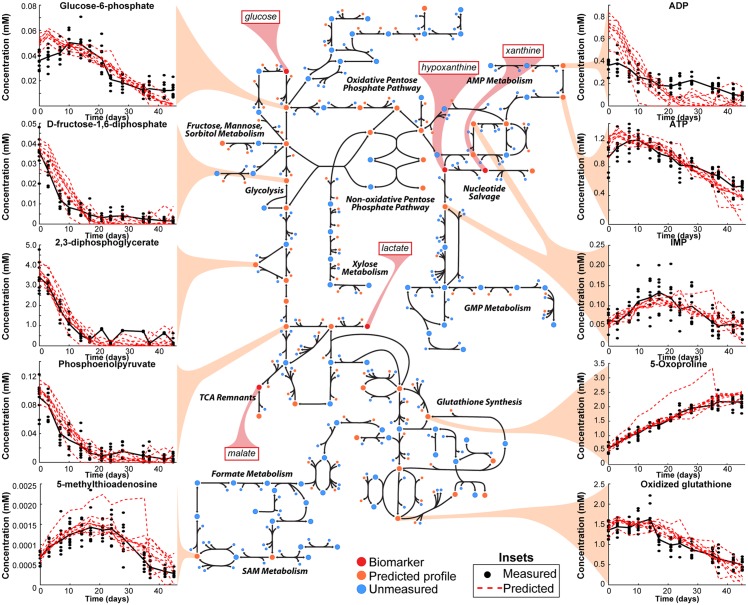
Predicted concentration profiles. Using the five biomarkers (highlighted in red), the concentration profiles for the remaining 91 measured metabolites were predicted (inset profile metabolites are highlighted in yellow). The remaining 81 predicted profiles are provided in the Supplementary Material. See S5 Fig for full map detail.

We tested the model’s capabilities by comparing the predicted profiles of the remaining 91 measured metabolites to their measured profiles (Fig 2). We calculated the symmetric mean absolute percentage error (SMAPE) for each predicted concentration profile, resulting in a median error of 0.1340 ± 0.1505 (S4 Fig). See Supplementary Material for all predicted profiles. To further validate our model, we compared against 10,000 profiles generated using a naive random walk for each metabolite. The naive random walk model assumes that metabolite concentration changes over time are independent of each other and are normally distributed. The random walk is a widely used benchmark for dynamic forecasting models [20]. When a significant number (≥500/10,000, i.e., ≥5%) of random walks outperform a trained model for a metabolite, we conclude that the dynamics of that metabolite are indiscernible from noise for the data given (see Methods for details on the random walk comparison). Despite the complexity of RBC metabolism, we found that 84/91 (92%) of RBC metabolites were predicted more accurately than random walks using five biomarkers as input (*p* < 0.05).

In an effort to lend biological intuition to this surprising result, we viewed these results in the context of the complete RBC metabolic network (Fig 2, S5 Fig). The map highlights several points. First, the five biomarkers are largely distributed across key subsystems. Surprisingly, two biomarkers are adjacent in the network: xanthine and hypoxanthine. From inspection of the map, it becomes more intuitive that to unambiguously predict IMP levels (Fig 2), both biomarkers need to be quantitatively measured.

## Discussion

RBCs in storage undergo a series of morphological changes (commonly referred to as “storage lesion”) that become more pronounced throughout the storage process [1, 21, 22]. Recent studies have shown that blood transfused after being stored for longer than five weeks is associated with post-transfusion complications [23, 24], indicating the serious clinical implications of metabolic decay in transfused blood. With the recent identification of eight extracellular biomarkers that are able to define this decay, the field of transfusion medicine now has an opportunity to define the metabolic state of stored RBCs with just a few measurements. Thus, there is a need for predictive modeling methods that can extend the applicability of these biomarkers to provide deeper understanding of the metabolic state of RBCs collected and stored under blood banking conditions using current and future technologies (e.g., improved bags or storage solutions, pathogen reduction technologies).

In this study, we have developed a statistical model that uses these biomarkers to predict the time series concentration profiles of other metabolites in the RBC metabolic network. This powerful tool was rigorously validated to avoid overfitting through model (complexity) and feature selection, and comparing against a standard forecasting baseline model (i.e., naive random walk). As with any data modeling approach, the performance of a model is dependent upon the quality of the input data; this is no exception here. We see that certain metabolites (e.g., ADP, inosine) had higher prediction errors, which can be partially attributed to noise in the training data and to low concentrations (S6 Fig).

The results presented here have two important implications. First, we have shown that if good biomarkers are available for a given system (like for the human RBC), then they can be used to make quantitative predictions about systemic behavior. Second, this provides the potential for a cost-effective workflow to monitor the metabolic state of a biological system since the only input under new conditions is the concentration profiles of biomarkers. Through the use of modeling and statistical analysis, the measured and predicted concentrations would enable a quantitative understanding of systems-level behavior.

Thus, we have demonstrated the predictive power of biomarkers through the use of a statistical model for RBCs in storage. This data-driven statistical modeling approach performed remarkably well for the RBC system, even without a detailed kinetic model. These results are encouraging and provide a complementary approach for predicting metabolite dynamics in less characterized organisms. As our validation procedure indicates, a critical mass of high-quality data is required to extract meaningful signals from noise. Our workflow provides a valuable assessment on whether this critical mass has been satisfied; the results here indicate that as few as 20 biological replicates are sufficient to provide a training set capable of achieving >90% accuracy. Follow up studies should address the question of how many measurements need to be made during storage in order to provide a reliable assessment of the RBC metabolome during storage, as this question has direct clinical implications.

As biomarkers are identified for new systems, there will be a need to analyze omics in an attempt to efficiently characterize complex biological systems using just these few informative measurements. Our workflow addresses this need by incorporating such biomarkers with a statistical model, offering broad utility in both the laboratory and the clinic.

## Methods

All computations were performed in Matlab R2016b (Mathworks, Natick, MA).

### System identification

An Output Error (OE) model [14] predicts system dynamics from past values, measured inputs, and unmeasured disturbances as follows:

$$y\left(t\right)=\sum_{i=1}^{n}\frac{B_{i}\left(q\right)}{F_{i}\left(q\right)}u_{i}\left(t-nk_{i}\right)+e\left(t\right)$$ (1)

where *y*(*t*) is the output at time *t*, *u*_*i*_ is an input (i.e., metabolite *i*), *e* is the unmeasured disturbance (i.e., system noise), and *B*(*q*) and *F*(*q*) are polynomials expressed in the time-shift operator *q* as follows:

$$B\left(q\right)=b_{1}+b_{2}q^{-1}+\ldots +b_{nb}q^{-nb+1}$$ (2)

$$F\left(q\right)=1+f_{1}q^{-1}+\ldots +f_{nf}q^{-nf}.$$ (3)

For this system, *n* = 5 (i.e., the five biomarkers), *nb* = 1, *nf* = 0, and there was no input delay (*nk* = 0). The *B* and *F* polynomials are estimated during the system identification step using least squares regression to minimize the difference between the measured signal and the predicted output.

This OE model performed better than more complex OE models having higher *nb* and more complex polynomial models. It also performed better than simpler linear regression—the OE model thus represents an optimal degree of complexity.

### Model evaluation

In order to evaluate the accuracy of the predicted concentration profiles for the various metabolites, we calculated the symmetric mean absolute percentage error (SMAPE), given by:

$$\text{SMAPE}=\frac{1}{n}\sum_{t=1}^{n}\frac{|y_{t}-{\hat{y}}_{t}|}{y_{t}+{\hat{y}}_{t}}$$ (4)

where *n* is the number of time points, *y* is the measured concentration profile, and $\hat{y}$ is the predicted concentration profile. For the global statistics reported in S4 Fig, the mean of the SMAPE of the 10 predicted profiles is given.

### Quantitative biomarker selection

We trained the OE model using a recently published metabolomics data set of RBCs under storage conditions at 4°C with 20 biological replicates from Bordbar et al. [10]. In order to predict the concentration of target metabolites, we used the eight extracellular biomarkers [6] as input since they are highly representative of the qualitative behavior of the rest of the system. In order to determine if these biomarkers are also good quantitative predictors, we performed a 10-fold cross validation on the set of 10 samples used for training the model to verify the generalization performance of the trained model. We ran our cross validation on all 56 combinations of five biomarkers (i.e., 8 choose 5); the five selected biomarkers had a mean SMAPE of 10.33%, which was within 1% of the top performing set of five biomarkers. Thus, we used glucose, hypoxanthine, lactate, malate, and xanthine as the final set of biomarkers input to the OE model.

### Training an ensemble of models

We trained an ensemble of OE models using the five biomarker profiles and each of the 91 measured metabolite profiles. Thus, we trained 91 ensemble models (one ensemble for each metabolite). Each ensemble model consisted of 10 OE models, each trained on a biological replicate. We used Bags 1–10 as this training set. We combined the outputs of these 10 OE models into a single prediction for each metabolite by computing the median of the 10 predictions at each time point (S3 Fig). This ensemble modeling approach captures the biological variability inherent among the samples used for training.

### Predictions on testing data

We used Bags 11–20 as the testing data set. In order to assess the variability between the training and testing data, we performed a two-sample *t*-test at each time point for each metabolite. This showed that approximately 24% of the data rejected the null hypothesis (FDR-adjusted *p* < 0.05) that the two data sets came from the same distribution and also showed greater than a 20% difference in the mean concentrations at a given time point (S7 Fig). For each test replicate, the five biomarkers were input to the trained ensemble model.

### Comparison to naive random walk

In addition to the prediction error, as computed by SMAPE, we also evaluated our model by comparing its performance against a benchmark model. We chose as a benchmark the random walk model, which assumes that metabolite concentration changes over time are independent of each other and are normally distributed with zero mean. The random walk model is commonly used to benchmark dynamic forecasting models [20]. To ensure that the random walk was representative of the metabolite concentration changes, we estimated the standard deviation of random changes from all 10 testing replicates across all time points for each metabolite. We further ensured that the random walk was an appropriate benchmark by initializing with a realistic concentration. To do so, we randomly chose from the pool of the 10 measured starting points of the testing replicates for each metabolite.

We generated 10,000 of these random walk profiles for each metabolite. In order to compare these to our model predictions, our null hypothesis was that our trained model performed no better than the random profiles. We calculated the SMAPE for each of the random profiles and compared to the SMAPE for the predicted profiles; the given *p* value is the number of random profiles which had a lower SMAPE than the average of the predicted profiles for that metabolite.

## Supporting information

S1 Fig Biomarker profiles. The concentration profiles for the biomarkers are shown for the full 45 day time course with all 14 time points included.
(PDF)

S2 Fig Metabolites whose behavior at Day 31 indicates that the time point should be removed. A: The concentration profile for intracellular glucose does not show an increase at Day 31 that corresponds with the spike observed in extracellular glucose (S1 Fig). B: The concentration profiles of extracellular chloride and sodium show the same abnormal behavior as extracellular glucose at Day 31.
(PDF)

S3 Fig Example of individual replicate predictions. Each subplot represents one testing replicate of the 10 shown in Fig 2. The red swathe represents the spread of the predictions of each of the 10 trained models included in the ensemble model. The red dashed line is the median of the 10 trained models and the final output for each replicate. The black points represent the measured testing data.
(PDF)

S4 Fig Global statistics for all predicted metabolites. We calculated the mean of the symmetric mean absolute percentage error (SMAPE) for the 10 predicted concentration profiles for each metabolite.
(PDF)

S5 Fig Full map for the RBC metabolic network. (PDF)

S6 Fig Metabolites with poor model predictions. The metabolites shown are those for which the model predictions were not significantly better (*p* > 0.05) than the naive random walk. The distribution of SMAPEs for all ten predictions are shown on the right.
(PDF)

S7 Fig Statistical analysis between training and testing data sets. The color bar represents the percent difference between the means concentrations for each metabolite at each time point between the training and testing data sets. An “X” indicates that the distributions of the training and testing data were significantly different (two-sample *t*-test, FDR-adjusted *p* < 0.05).
(PDF)

S8 Fig Measured data and predicted profiles. The distribution of SMAPEs for all ten predictions are shown on the right. Abbreviations are BiGG metabolite IDs.
(PDF)

S9 Fig Measured data and predicted profiles. The distribution of SMAPEs for all ten predictions are shown on the right. Abbreviations are BiGG metabolite IDs.
(PDF)

S10 Fig Measured data and predicted profiles. The distribution of SMAPEs for all ten predictions are shown on the right. Abbreviations are BiGG metabolite IDs.
(PDF)

S11 Fig Measured data and predicted profiles. The distribution of SMAPEs for all ten predictions are shown on the right. Abbreviations are BiGG metabolite IDs.
(PDF)

S12 Fig Measured data and predicted profiles. The distribution of SMAPEs for all ten predictions are shown on the right. Abbreviations are BiGG metabolite IDs.
(PDF)

S13 Fig Measured data and predicted profiles. The distribution of SMAPEs for all ten predictions are shown on the right. Abbreviations are BiGG metabolite IDs.
(PDF)

S14 Fig Measured data and predicted profiles. The distribution of SMAPEs for all ten predictions are shown on the right. Abbreviations are BiGG metabolite IDs.
(PDF)

S15 Fig Measured data and predicted profiles. The distribution of SMAPEs for all ten predictions are shown on the right. Abbreviations are BiGG metabolite IDs.
(PDF)
