# Supplementary figures and images for: Biomarkers are used to predict quantitative metabolite concentration profiles in human red blood cells
Source: PLoS Comput Biol. 2017 Mar 6;13(3):e1005424. doi: 10.1371/journal.pcbi.1005424 (PMC5358888; doi:10.1371/journal.pcbi.1005424)

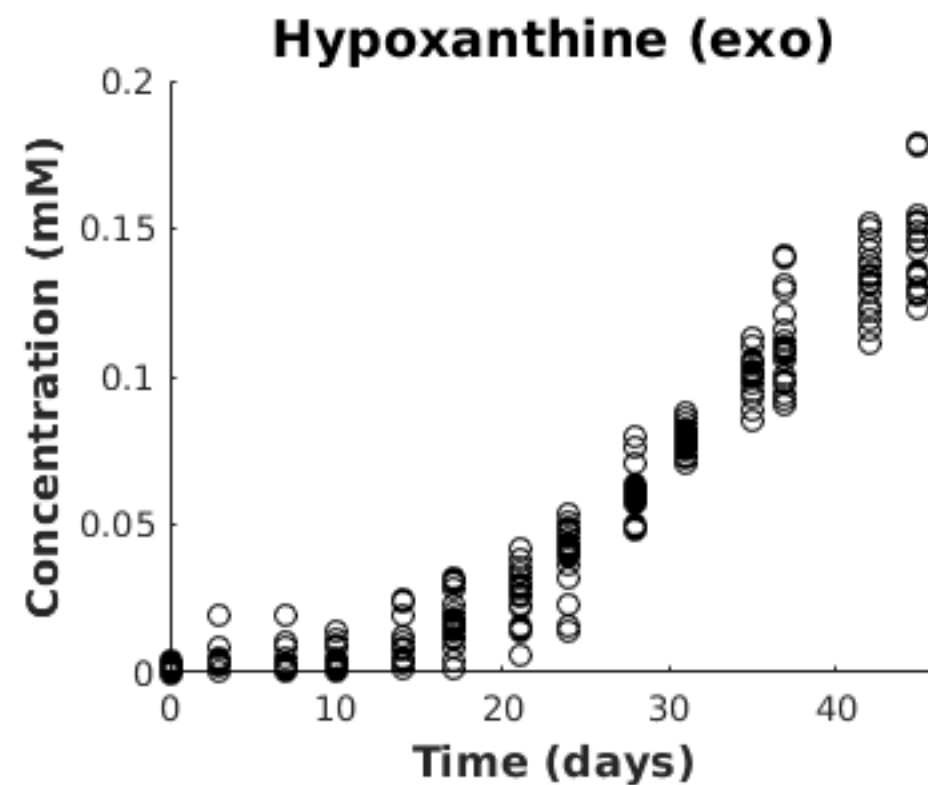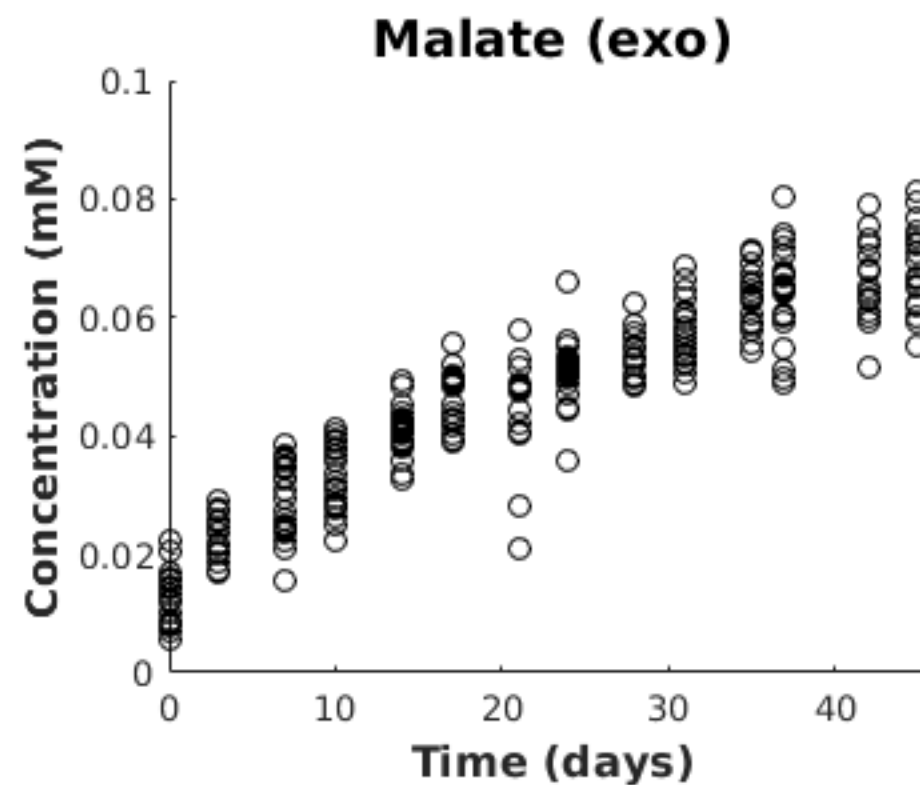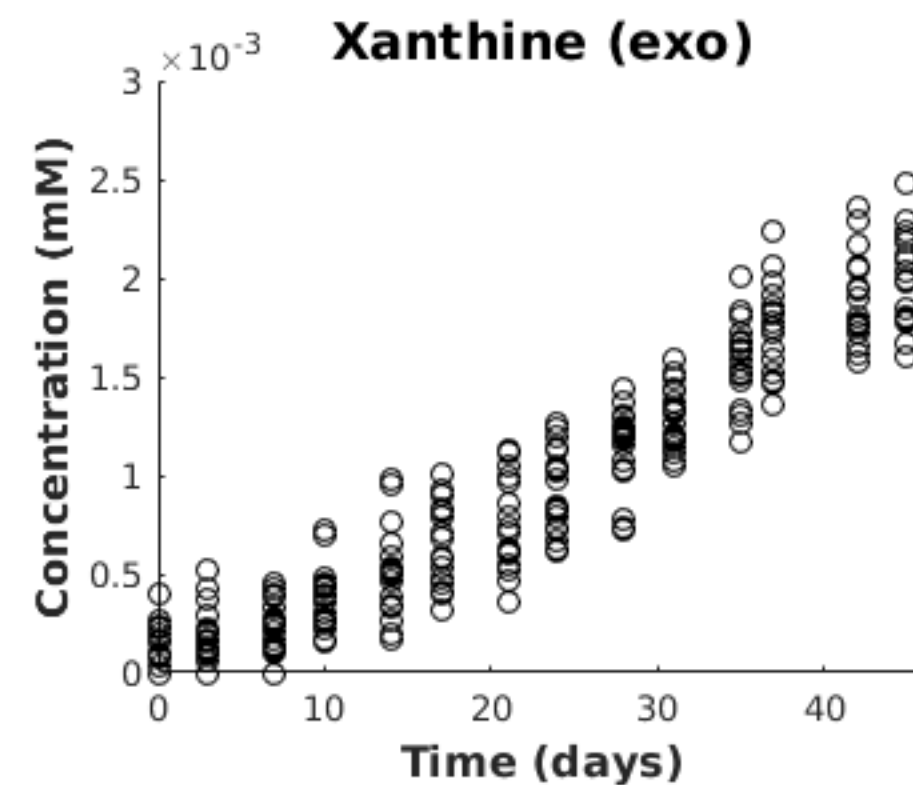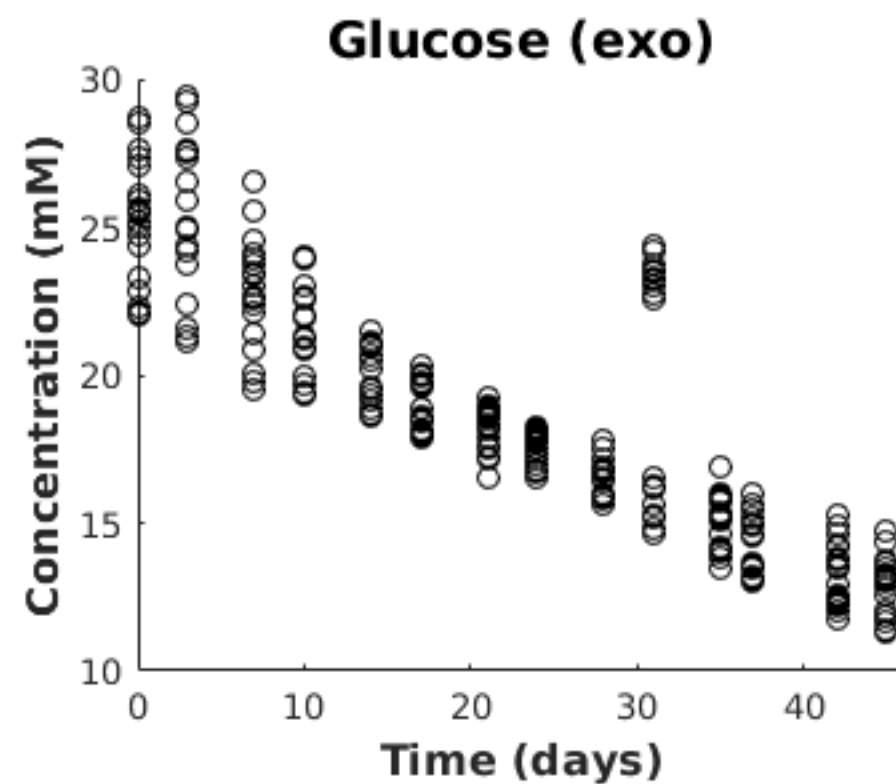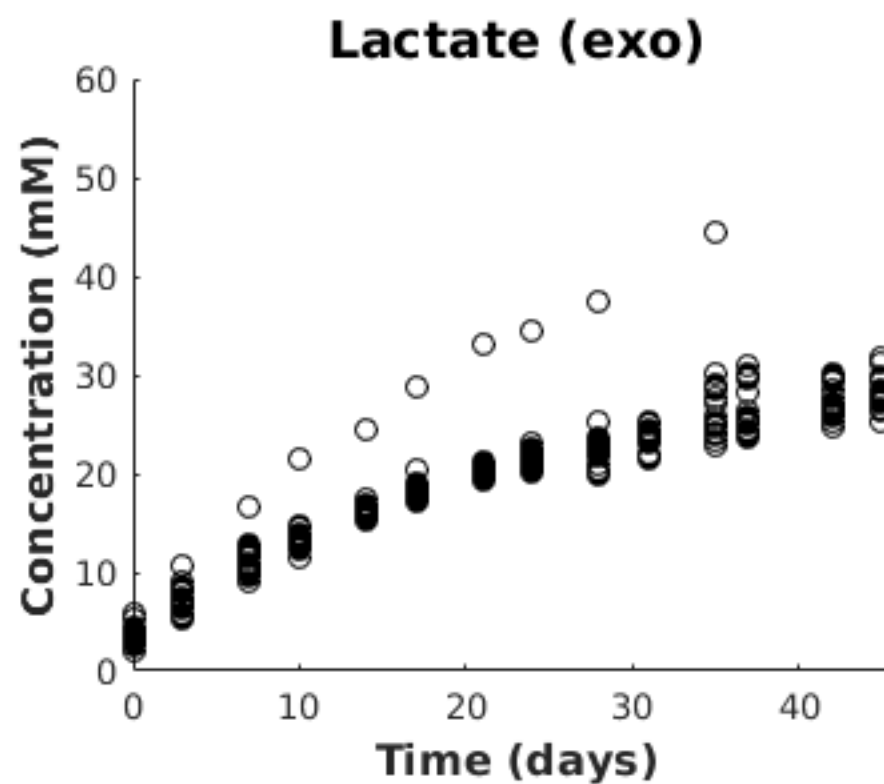

Supplement: S1 Fig — The concentration profiles for the biomarkers are shown for the full 45 day time course with all 14 time points included. (PDF) [file pcbi.1005424.s001.pdf]

**A**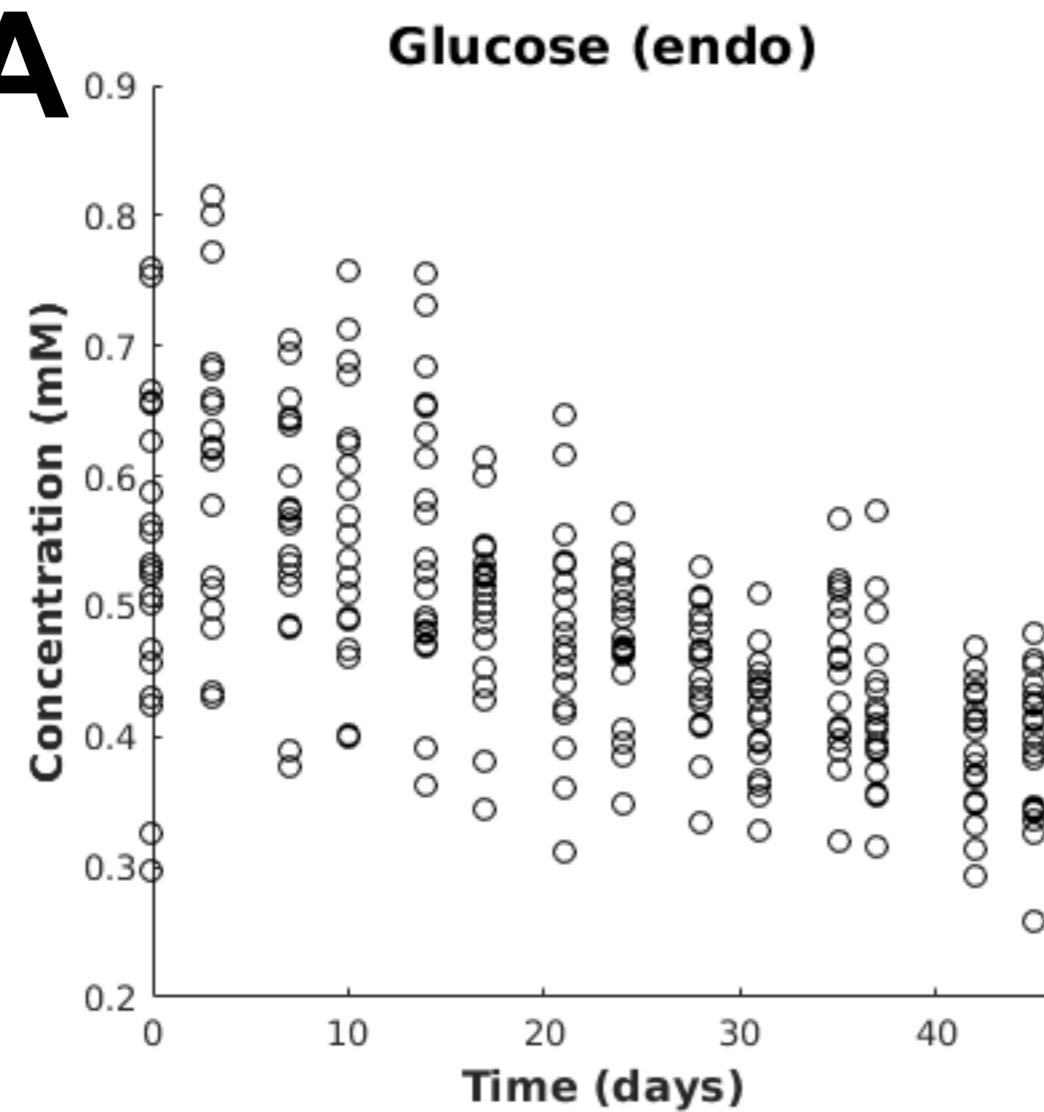**B**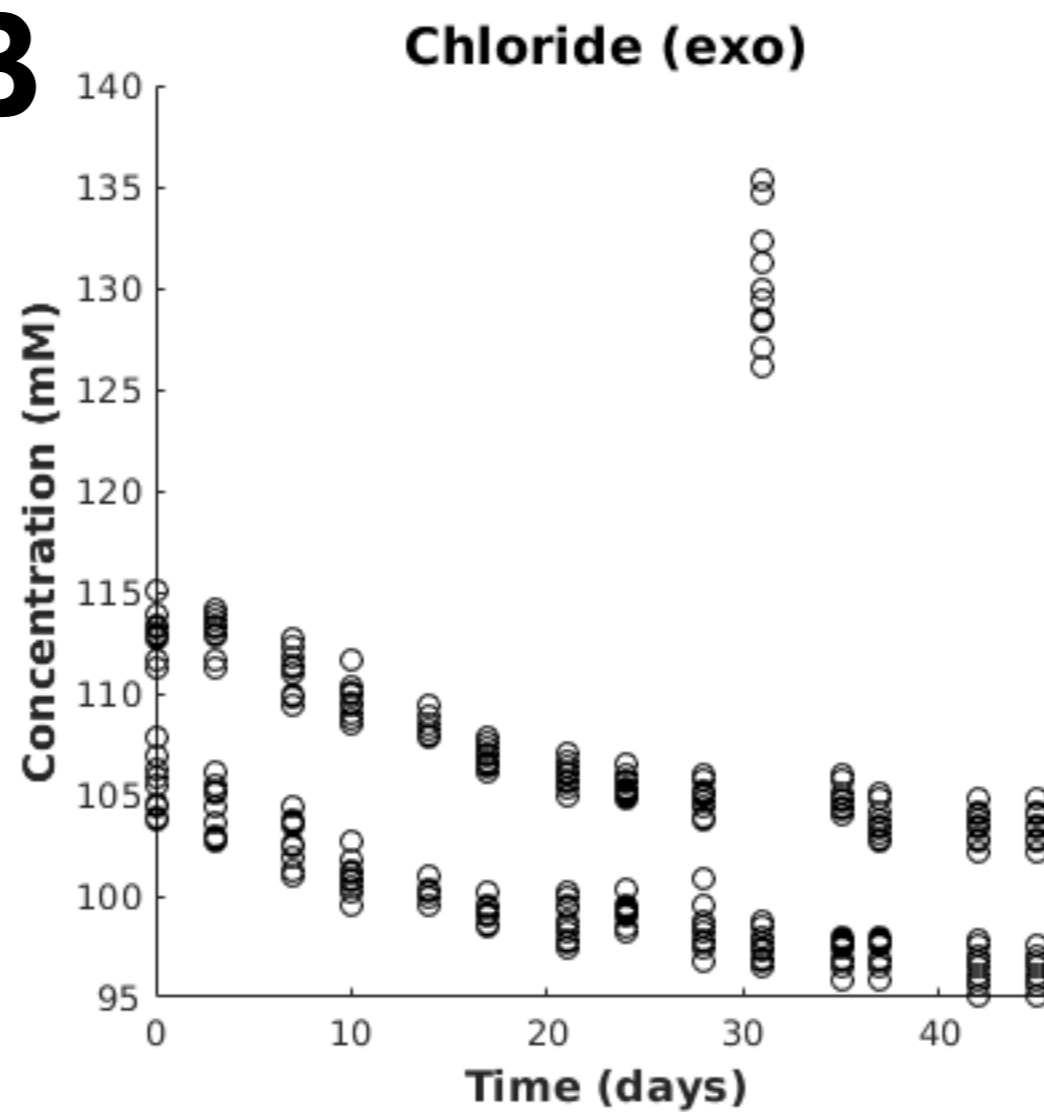**Sodium (exo)**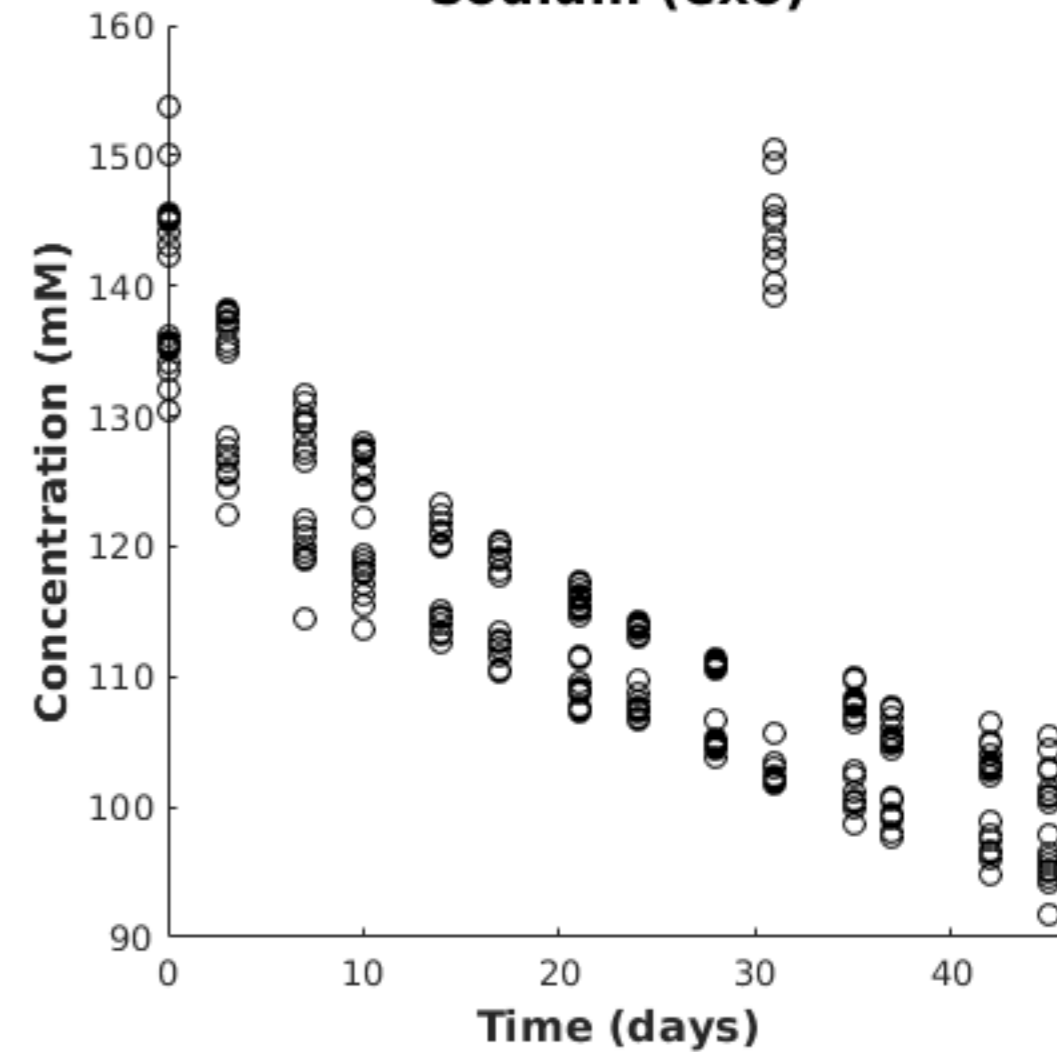

Supplement: S2 Fig — A: The concentration profile for intracellular glucose does not show an increase at Day 31 that corresponds with the spike observed in extracellular glucose (S1 Fig). B: The concentration profiles of extracellular chloride and sodium show the same abnormal behavior as extracellular glucose at Day 31. (PDF) [file pcbi.1005424.s002.pdf]

# Individual Replicate Predictions for fdp[c]

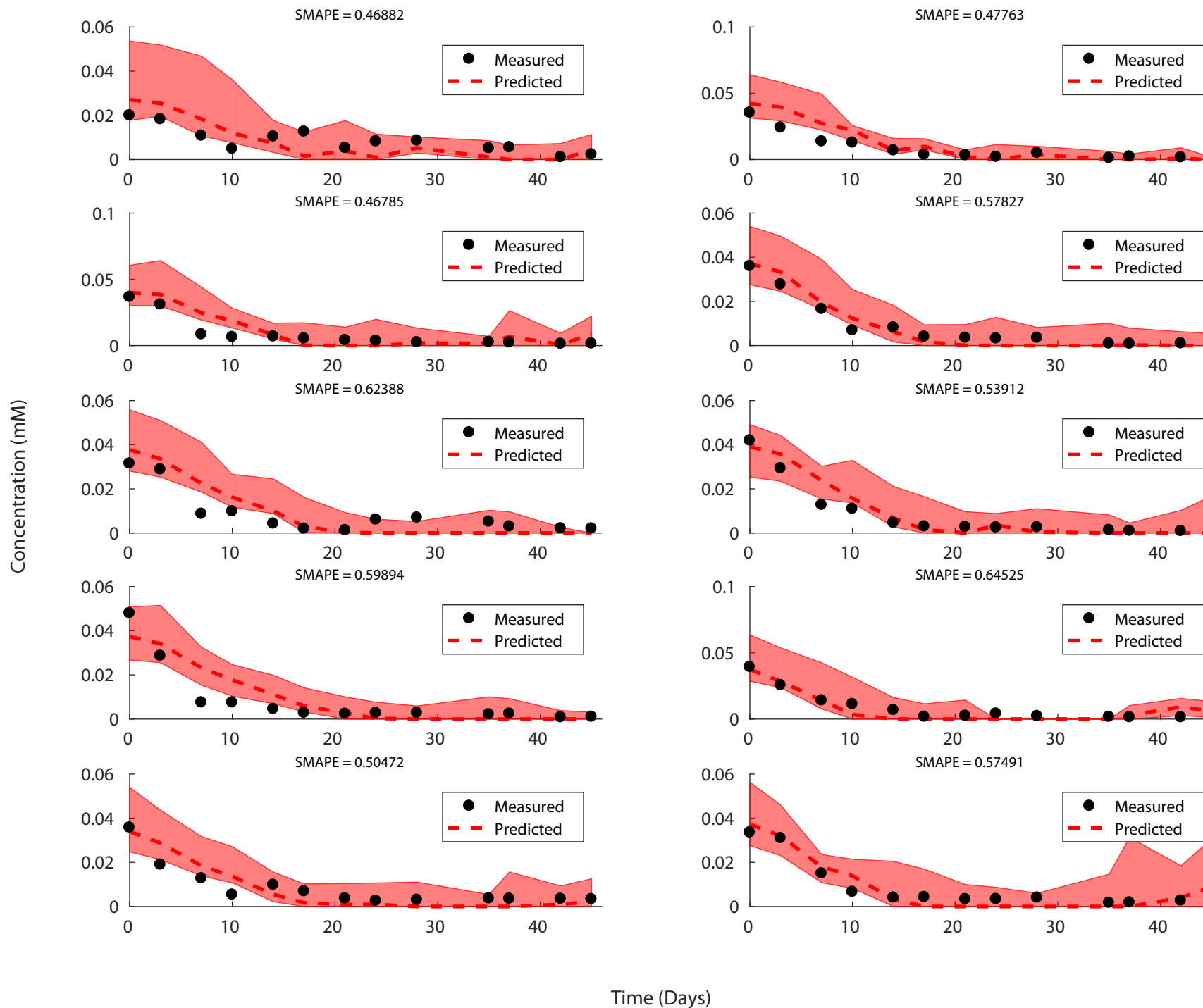

Supplement: S3 Fig — Each subplot represents one testing replicate of the 10 shown in Fig 2. The red swathe represents the spread of the predictions of each of the 10 trained models included in the ensemble model. The red dashed line is the median of the 10 trained models and the final output for each replicate. The black points represent the measured testing data. (PDF) [file pcbi.1005424.s003.pdf]

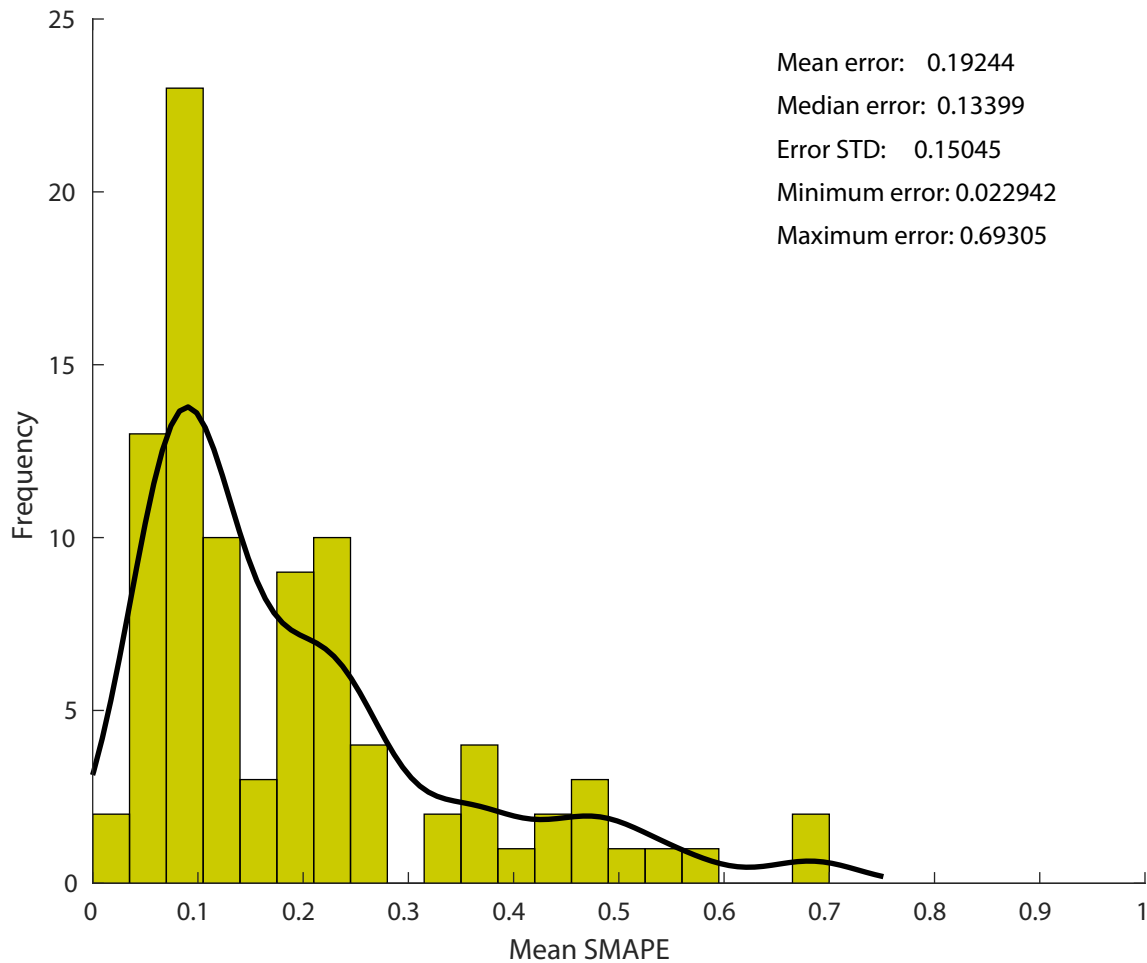

Supplement: S4 Fig — We calculated the mean of the symmetric mean absolute percentage error (SMAPE) for the 10 predicted concentration profiles for each metabolite. (PDF) [file pcbi.1005424.s004.pdf]

RBC Metabolic Network

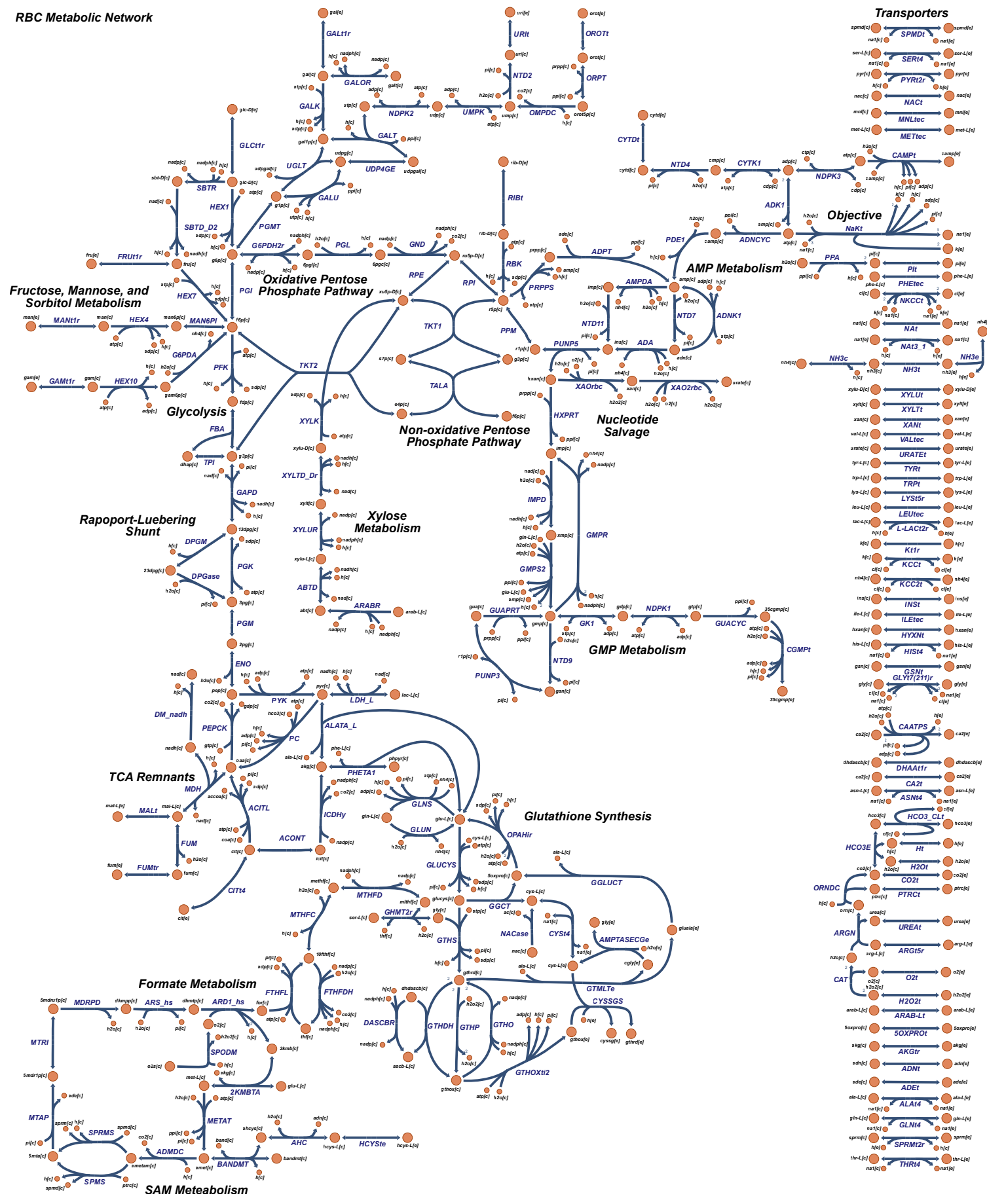

Supplement: S5 Fig — (PDF) [file pcbi.1005424.s005.pdf]

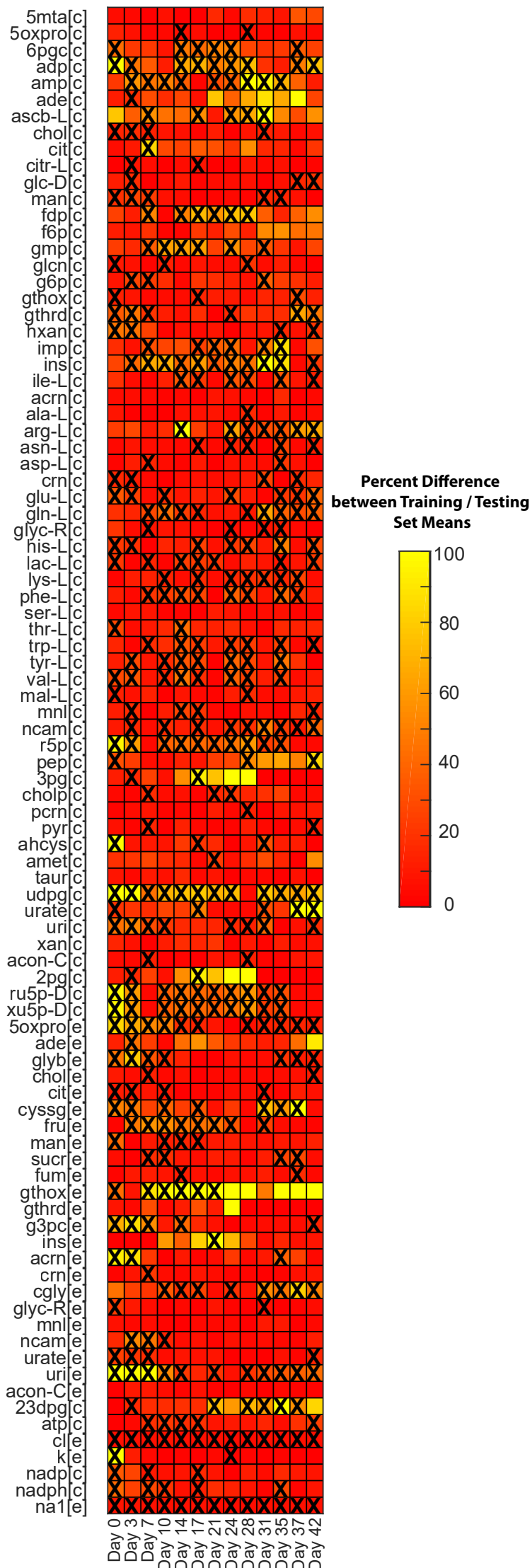

Supplement: S7 Fig — The color bar represents the percent difference between the means concentrations for each metabolite at each time point between the training and testing data sets. An “X” indicates that the distributions of the training and testing data were significantly different (two-sample t-test, FDR-adjusted p < 0.05). (PDF) [file pcbi.1005424.s007.pdf]

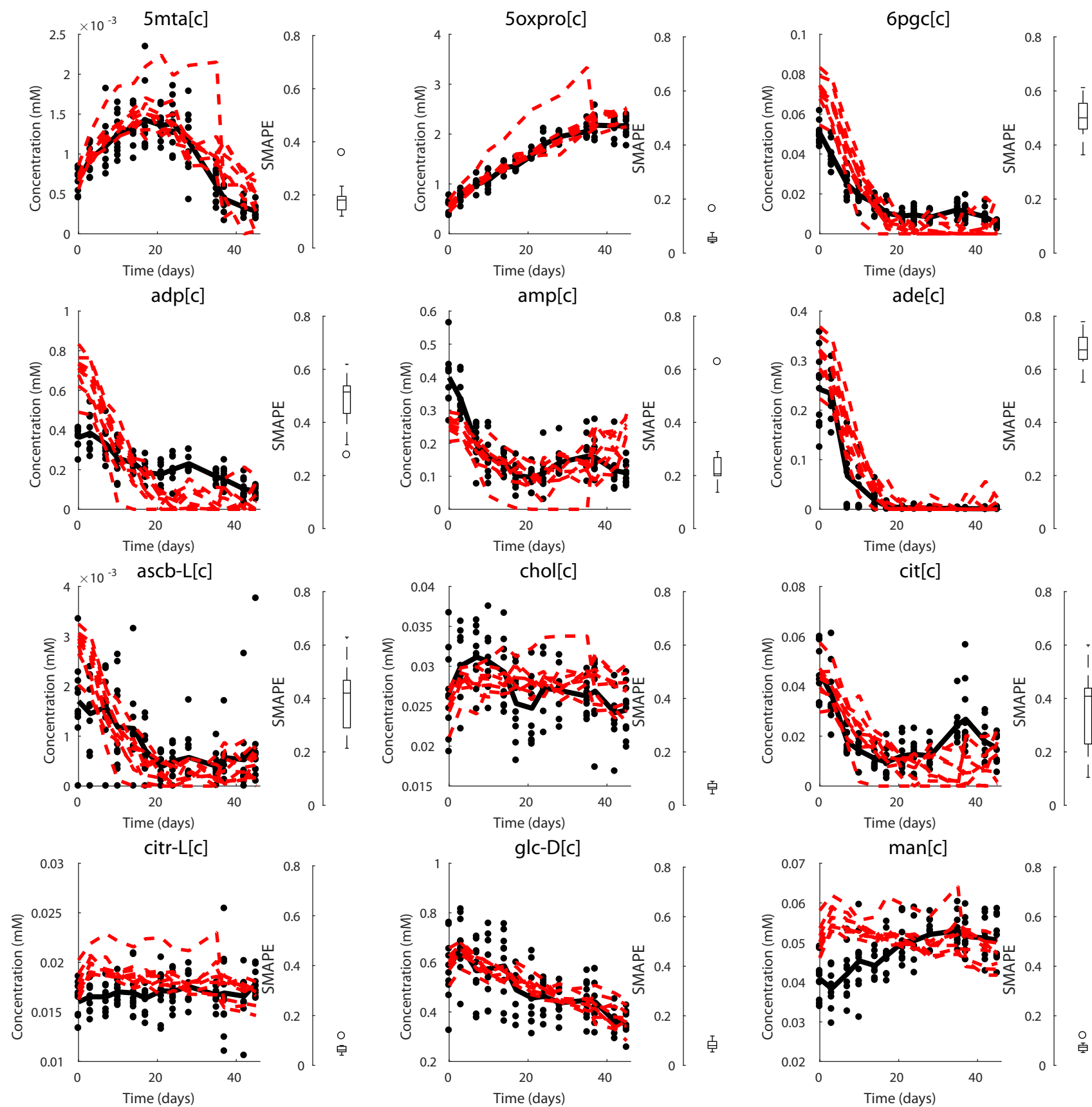

● Measured — Predicted

Supplement: S8 Fig — The distribution of SMAPEs for all ten predictions are shown on the right. Abbreviations are BiGG metabolite IDs. (PDF) [file pcbi.1005424.s008.pdf]

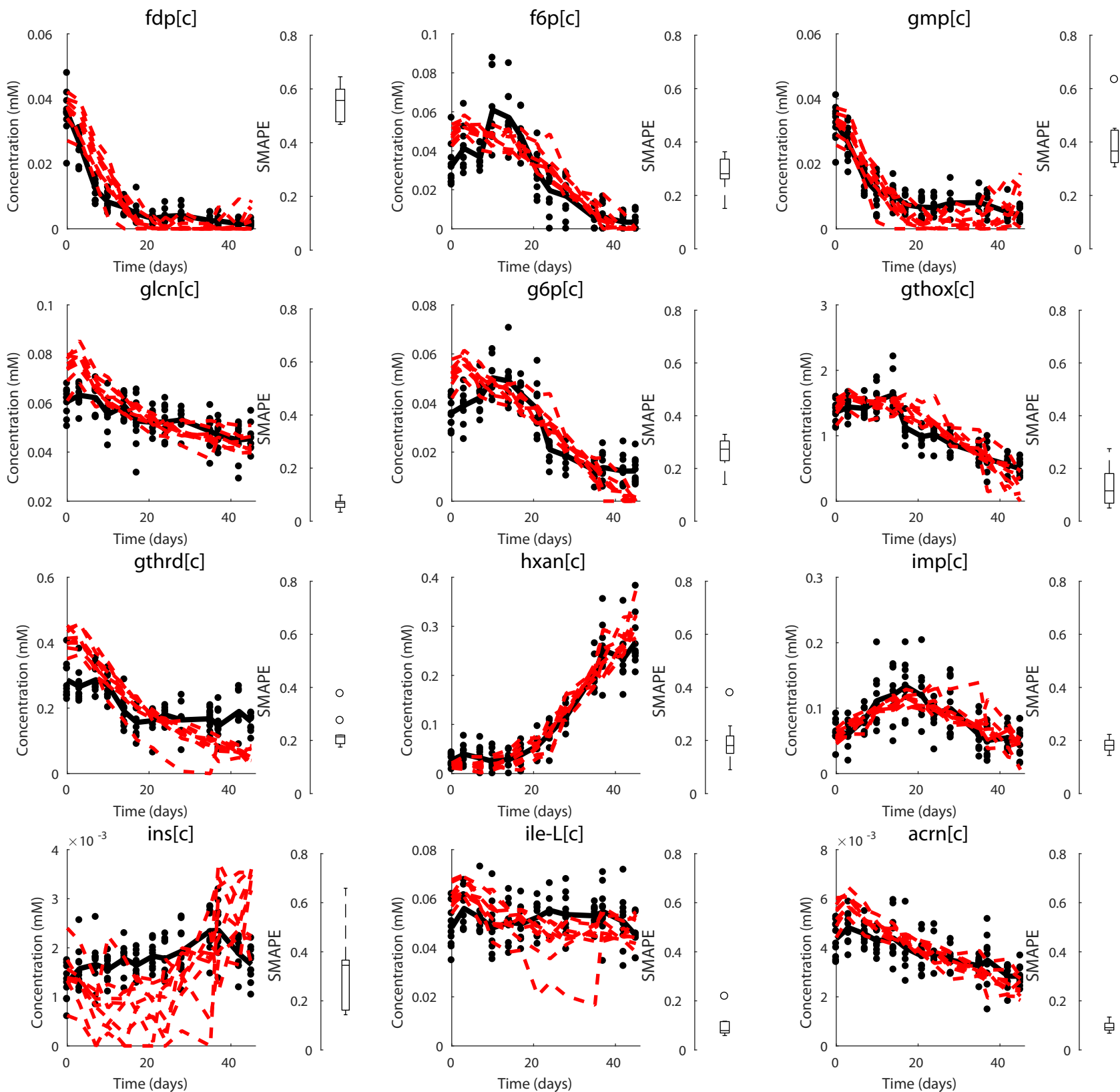

● Measured — Predicted

Supplement: S9 Fig — The distribution of SMAPEs for all ten predictions are shown on the right. Abbreviations are BiGG metabolite IDs. (PDF) [file pcbi.1005424.s009.pdf]

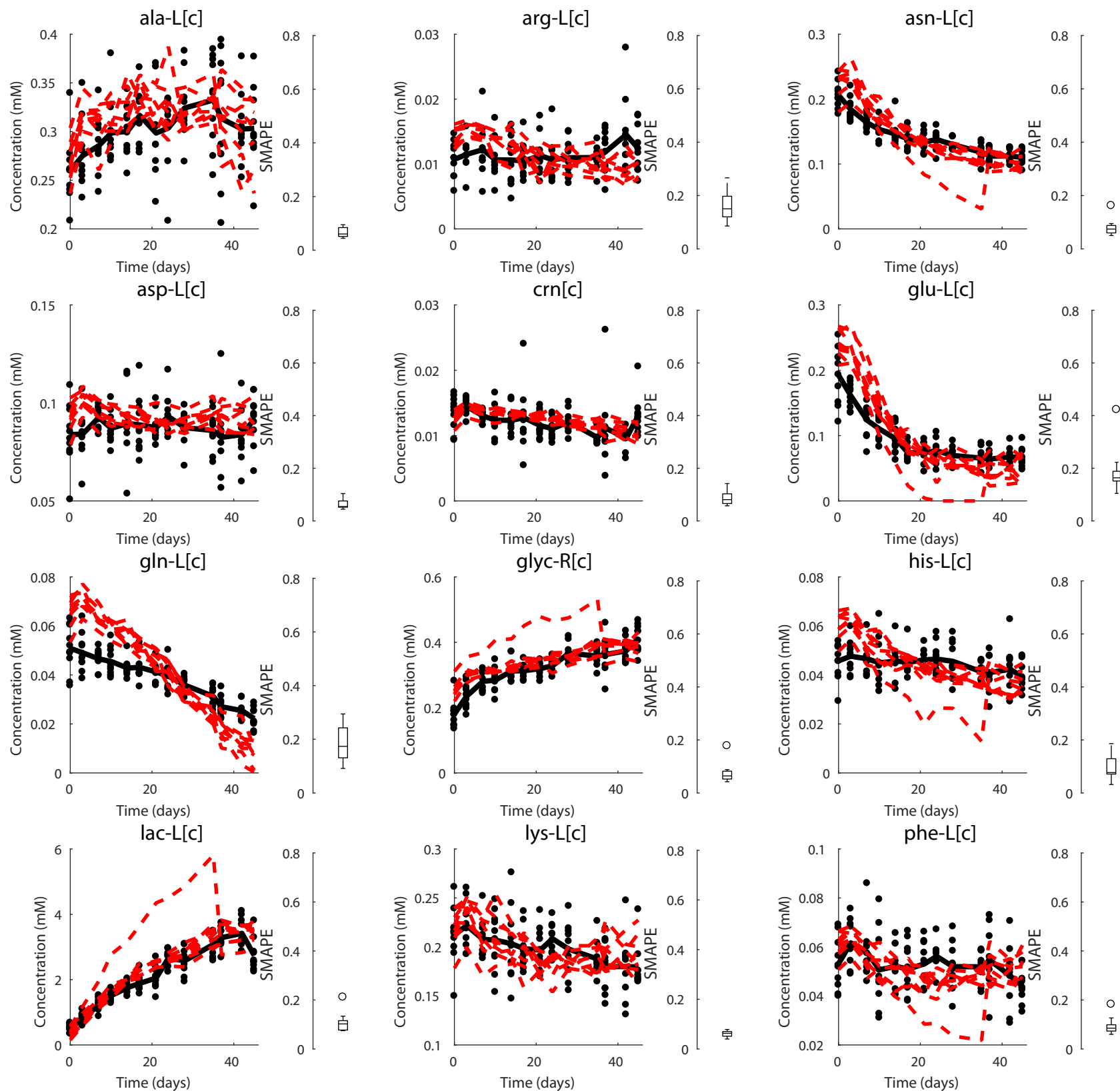

● Measured — Predicted

Supplement: S10 Fig — The distribution of SMAPEs for all ten predictions are shown on the right. Abbreviations are BiGG metabolite IDs. (PDF) [file pcbi.1005424.s010.pdf]

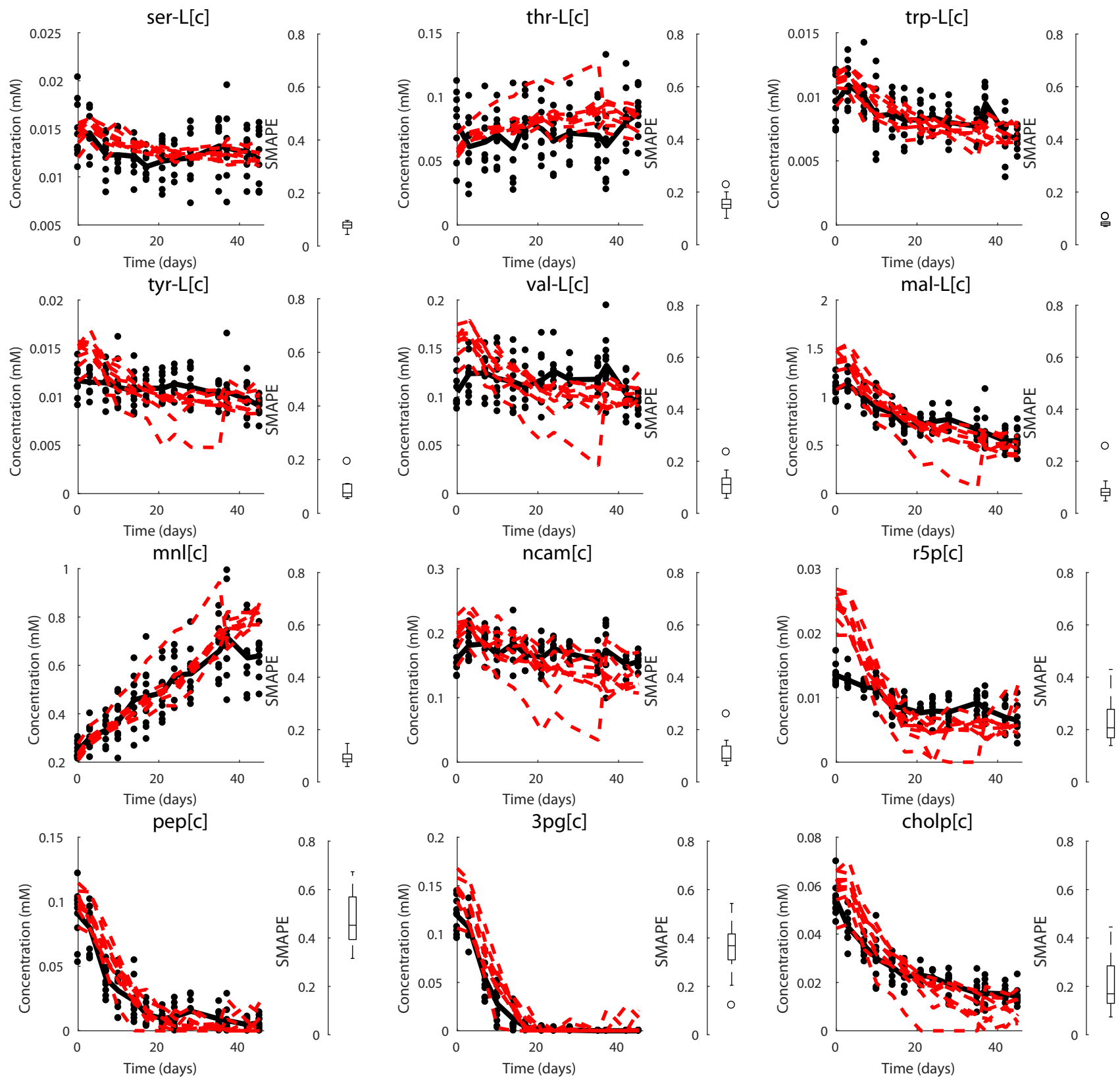

● Measured — Predicted

Supplement: S11 Fig — The distribution of SMAPEs for all ten predictions are shown on the right. Abbreviations are BiGG metabolite IDs. (PDF) [file pcbi.1005424.s011.pdf]

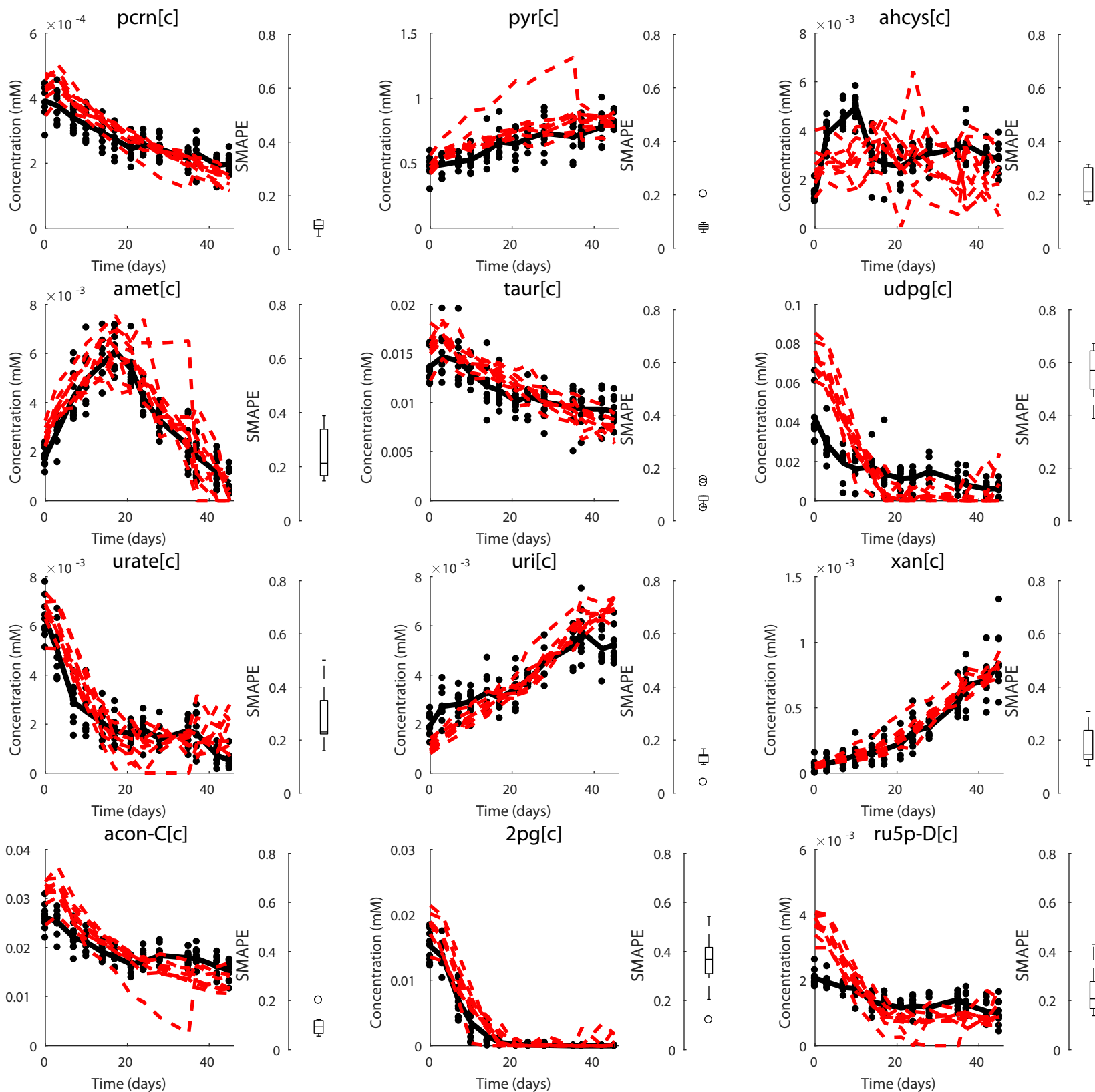

● Measured — Predicted

Supplement: S12 Fig — The distribution of SMAPEs for all ten predictions are shown on the right. Abbreviations are BiGG metabolite IDs. (PDF) [file pcbi.1005424.s012.pdf]

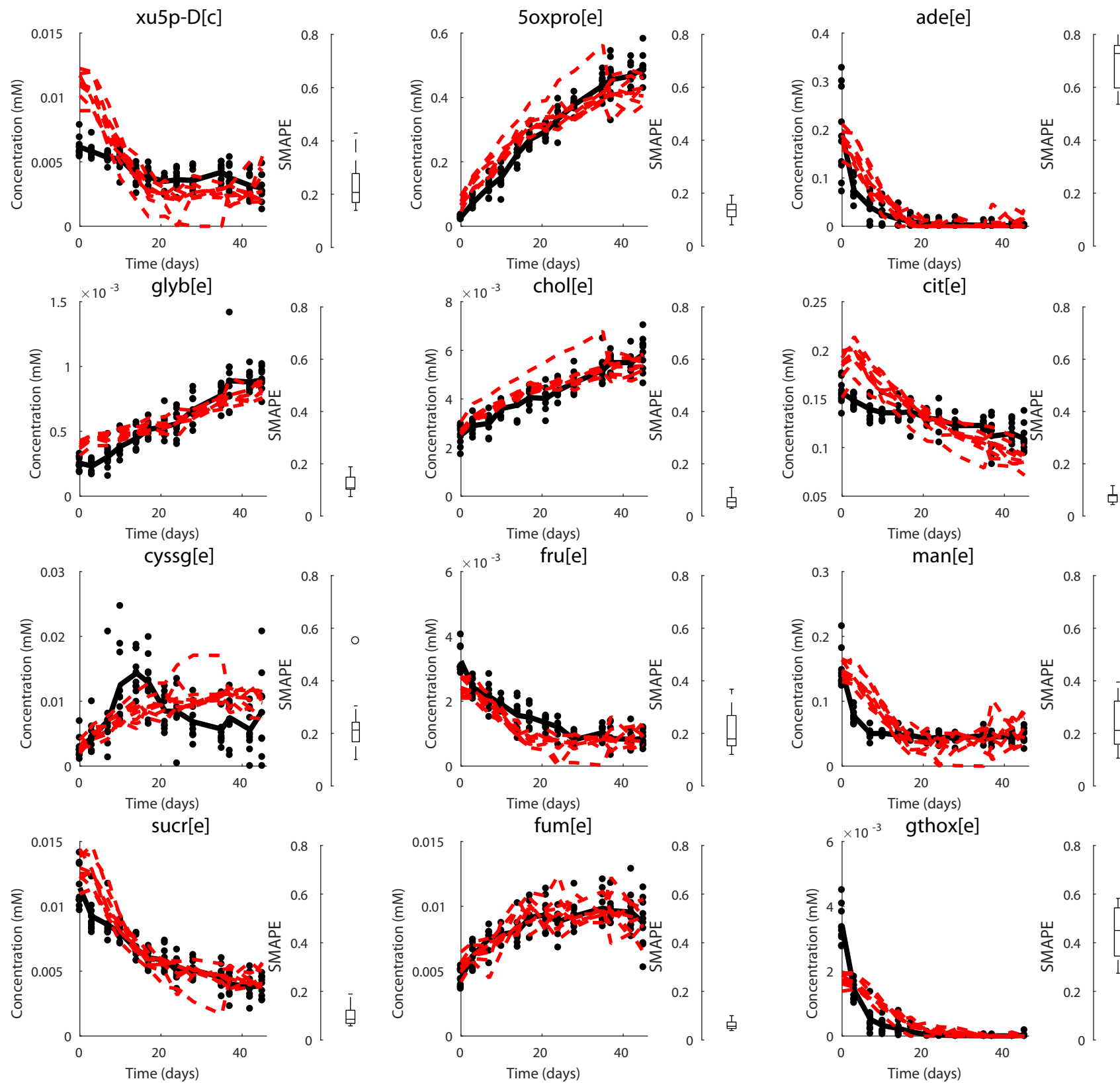

● Measured — Predicted

Supplement: S13 Fig — The distribution of SMAPEs for all ten predictions are shown on the right. Abbreviations are BiGG metabolite IDs. (PDF) [file pcbi.1005424.s013.pdf]

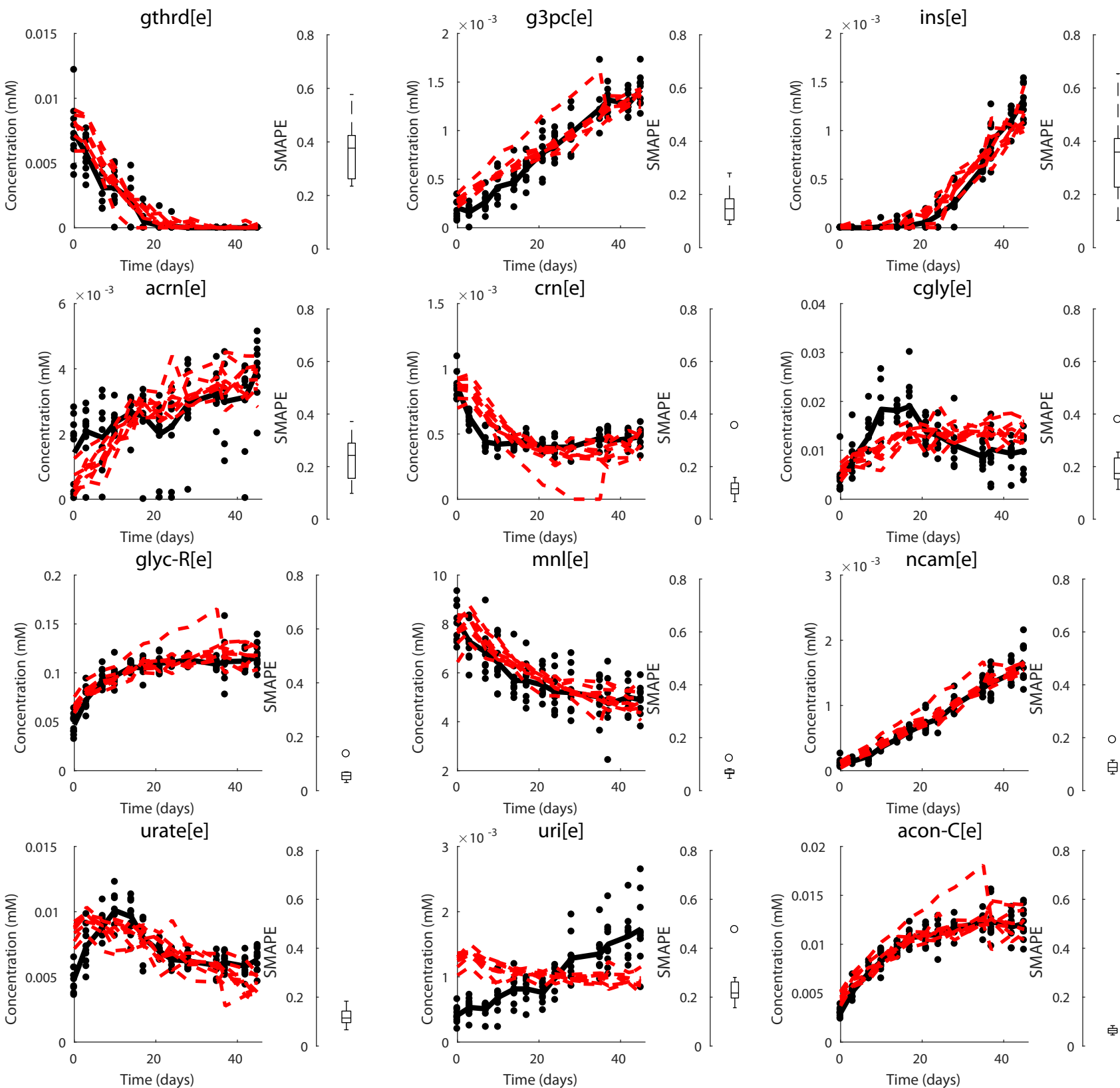

● Measured — Predicted

Supplement: S14 Fig — The distribution of SMAPEs for all ten predictions are shown on the right. Abbreviations are BiGG metabolite IDs. (PDF) [file pcbi.1005424.s014.pdf]

23dpg[c]

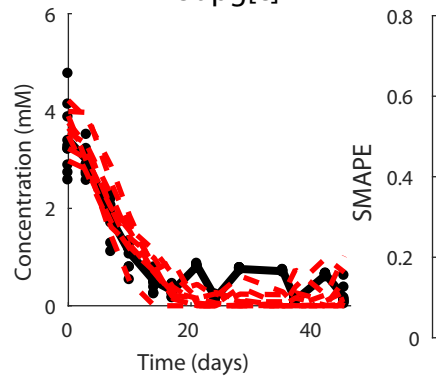

atp[c]

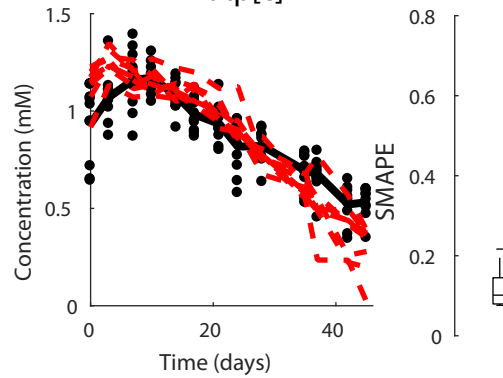

cl[e]

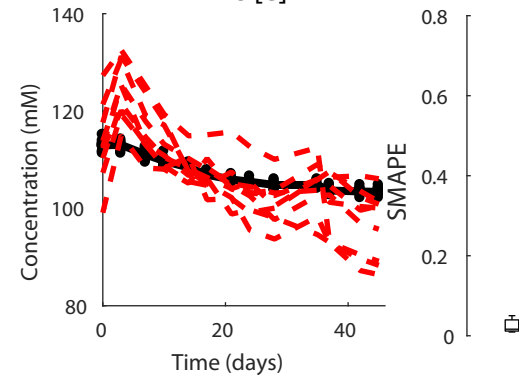

k[e]

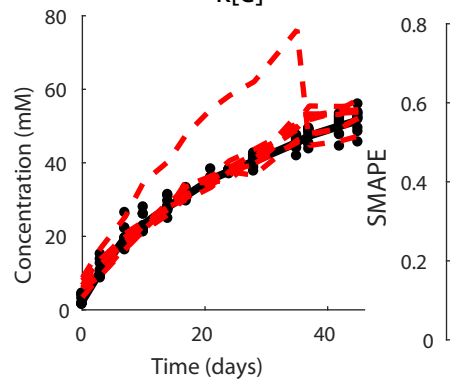

nadp[c]

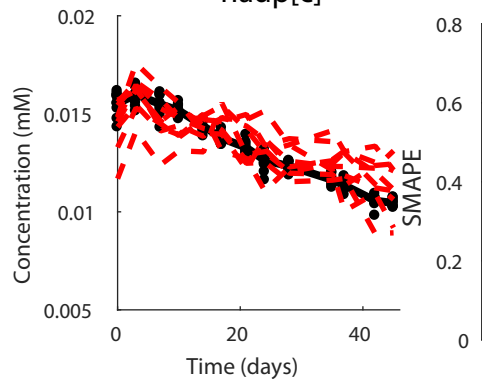

nadph[c]

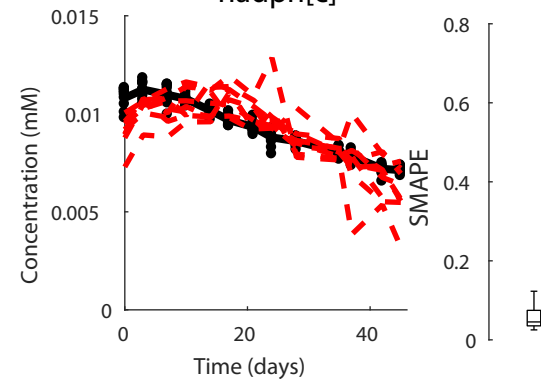

na1[e]

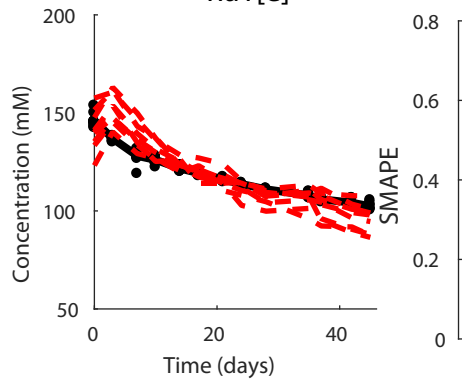

● Measured — Predicted

Supplement: S15 Fig — The distribution of SMAPEs for all ten predictions are shown on the right. Abbreviations are BiGG metabolite IDs. (PDF) [file pcbi.1005424.s015.pdf]
