# Supplementary material for: Biomarkers are used to predict quantitative metabolite concentration profiles in human red blood cells
Source: PLoS Comput Biol. 2017 Mar 6;13(3):e1005424. doi: 10.1371/journal.pcbi.1005424 (PMC5358888; doi:10.1371/journal.pcbi.1005424)
Supplement: S6 Fig — The metabolites shown are those for which the model predictions were not significantly better (p > 0.05) than the naive random walk. The distribution of SMAPEs for all ten predictions are shown on the right. (PDF) [file pcbi.1005424.s006.pdf]

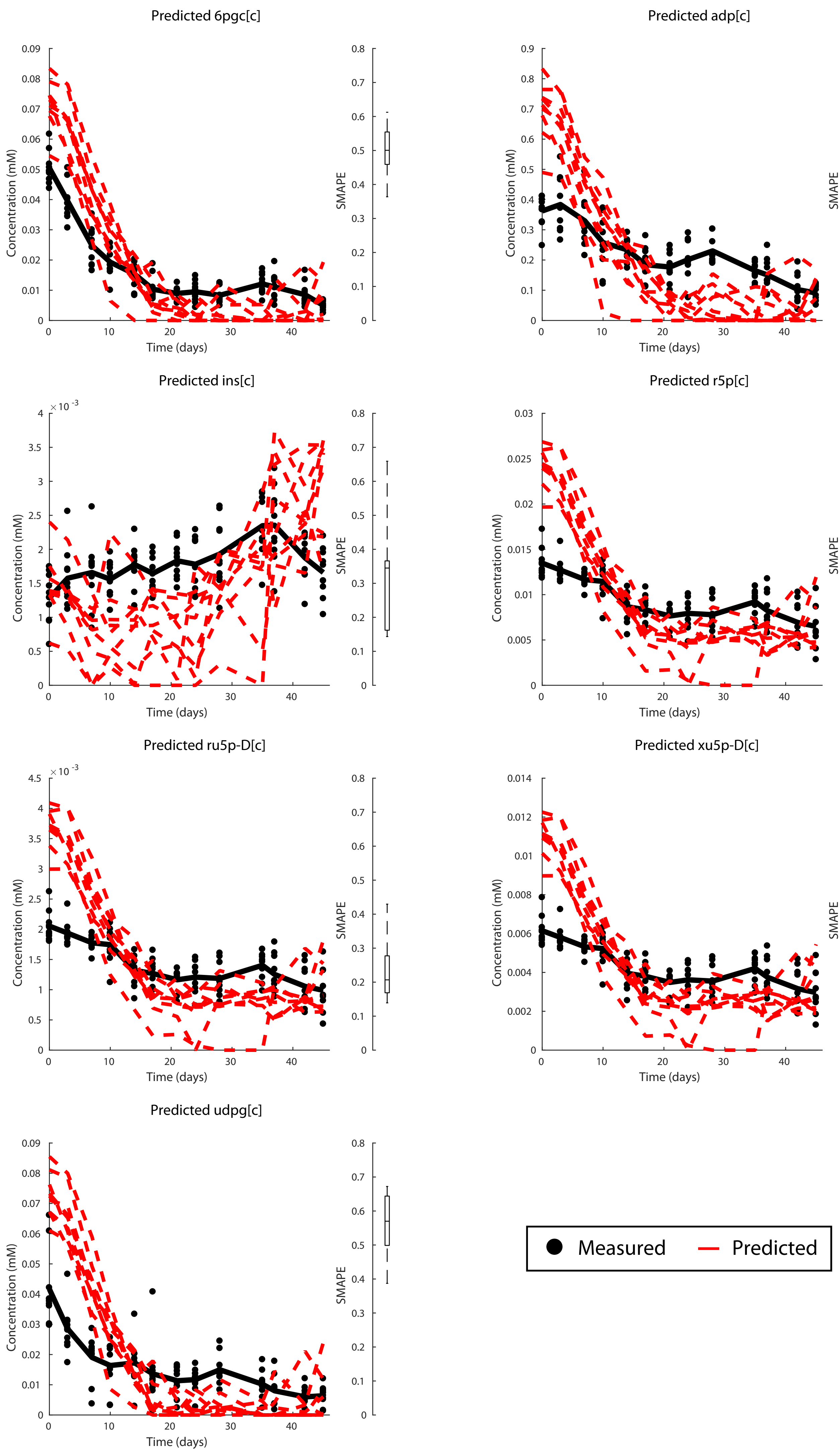
Predicted 6pgc[c]
Predicted adp[c]
Predicted ins[c]
Predicted r5p[c]
Predicted ru5p-D[c]
Predicted xu5p-D[c]
Predicted udpg[c]
Concentration (mM)
Time (days)
SMAPE
Measured
Predicted
